# Cesarean Scar Ectopic Pregnancy: A Diagnostic and Management Challenge

**DOI:** 10.7759/cureus.14463

**Published:** 2021-04-13

**Authors:** Koulshan Jameel, Gul-e-rana Abdul Mannan, Rabiya Niaz, Durr-e-shahwar Hayat

**Affiliations:** 1 Obstetrics and Gynecology, Canadian Specialist Hospital, Dubai, ARE; 2 Radiology, Dr. Ziauddin University Hospital, Karachi, PAK

**Keywords:** cesarean scar, ectopic pregnancy

## Abstract

Cesarean section scar pregnancy is the rarest form of ectopic pregnancy. Cesarean scar ectopic pregnancy poses a diagnostic and management challenge, and if not diagnosed and adequately treated in early pregnancy, it may lead to considerable maternal morbidity or mortality. We describe the presentation, workup including radiology studies, and subsequent management plan of a cesarean scar ectopic pregnancy in a 34-year-old female with a history of four previous cesarean sections. We were successful in treating this rare form of ectopic pregnancy without any maternal morbidity with a combination of medical and surgical management.

## Introduction

With the increase in the incidence of cesarean section, any subsequent risks of placenta previa, placenta accreta, and ectopic pregnancy have increased. Though rare, cesarean ectopic pregnancy has also increased in parallel with increase in cesarean rates. Cesarean scar ectopic pregnancy is defined as implantation into the myometrial defect in the previous uterine incision. The prevalence of cesarean ectopic pregnancy is estimated to be one in 2,000 pregnancies, which could be potential viable pregnancies or miscarriages into the scar [1]. Vial et al. [2] proposed that there are two different kinds of cesarean scar ectopic pregnancies, one that grows inside into the uterine cavity as gestational sac develops and has the potential to reach viable gestation but with risk of placenta accreta and major obstetric hemorrhage-the endogenous type. The other type is exogenous, which grows outward toward the bladder with potential for scar rupture and intra-abdominal bleeding. In such cases, early detection is of utmost importance to decrease the complications associated with it [3].

## Case presentation

**Figure 1:**
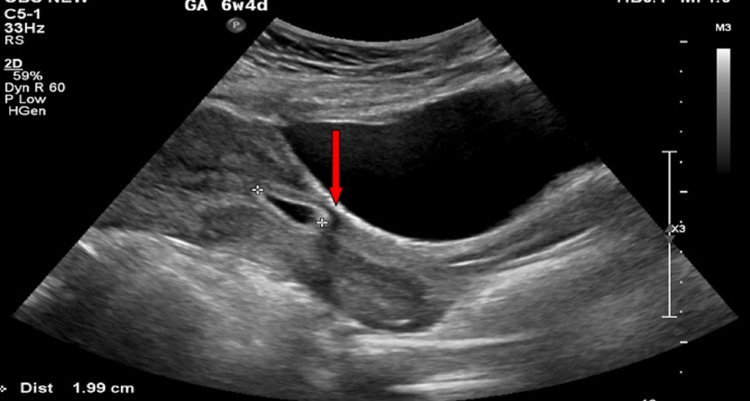
Transabdominal ultrasound showing 6 mm single small gestational sac with yolk sac of five weeks located in anterior part of lower uterine segment at cesarean scar, myometrium between bladder wall and gestational sac absent, and empty uterine cavity shown by red arrow.

The patient is a 34-year-old Para-4, Abortion-1, presented to outpatient clinic for contraceptive advice. Her past history is significant for four previous cesarean sections, the last one done eight months ago. She was lactating and having irregular periods. On her presentation in our clinic, general physical examination was unremarkable. Beta human chorionic gonadotropin (β-hCG) was positive at 915 mIU/mL. Ultrasound was not confirmative of pregnancy, and so β-hCG was repeated after 48 hours and showed doubling titer. She was advised for close follow-up later to check for fetal viability. In the next visit after one week, she complained of mild lower abdominal pain. Transabdominal ultrasound was done that showed 6 mm single small gestational sac with yolk sac of five weeks located in the anterior part of lower uterine segment at cesarean scar (Figure 1); myometrium between bladder wall and gestational sac was absent; uterine cavity was empty; findings were consistent with cesarean scar ectopic pregnancy. After confirmation of the diagnosis, plan of management was discussed with patient. Conservative management, medical management with systemic or local methotrexate injection, and surgical management including hysteroscopic suction evacuation/resection or laparoscopic wedge resection were discussed with the patient. Departmental meeting agreed on combining medical and surgical options. It was planned to proceed with systemically given intramuscular methotrexate injection, then followed after one week with hysteroscopic suction evacuation of products of conception. Measures to decrease blood loss during the procedure included preparation of blood and blood products and ready availability of balloon tamponade catheters in case bleeding ensues. Option of uterine artery embolization for reduction of blood loss was not accepted by the patient due to its associated risks. General surgeon was involved in case laparoscopic assistance required. Informed written consent was obtained from the patient informing her the risk of massive bleeding, need for laparotomy, and requirement of hysterectomy if bleeding does not get controlled.

**Figure 2:**
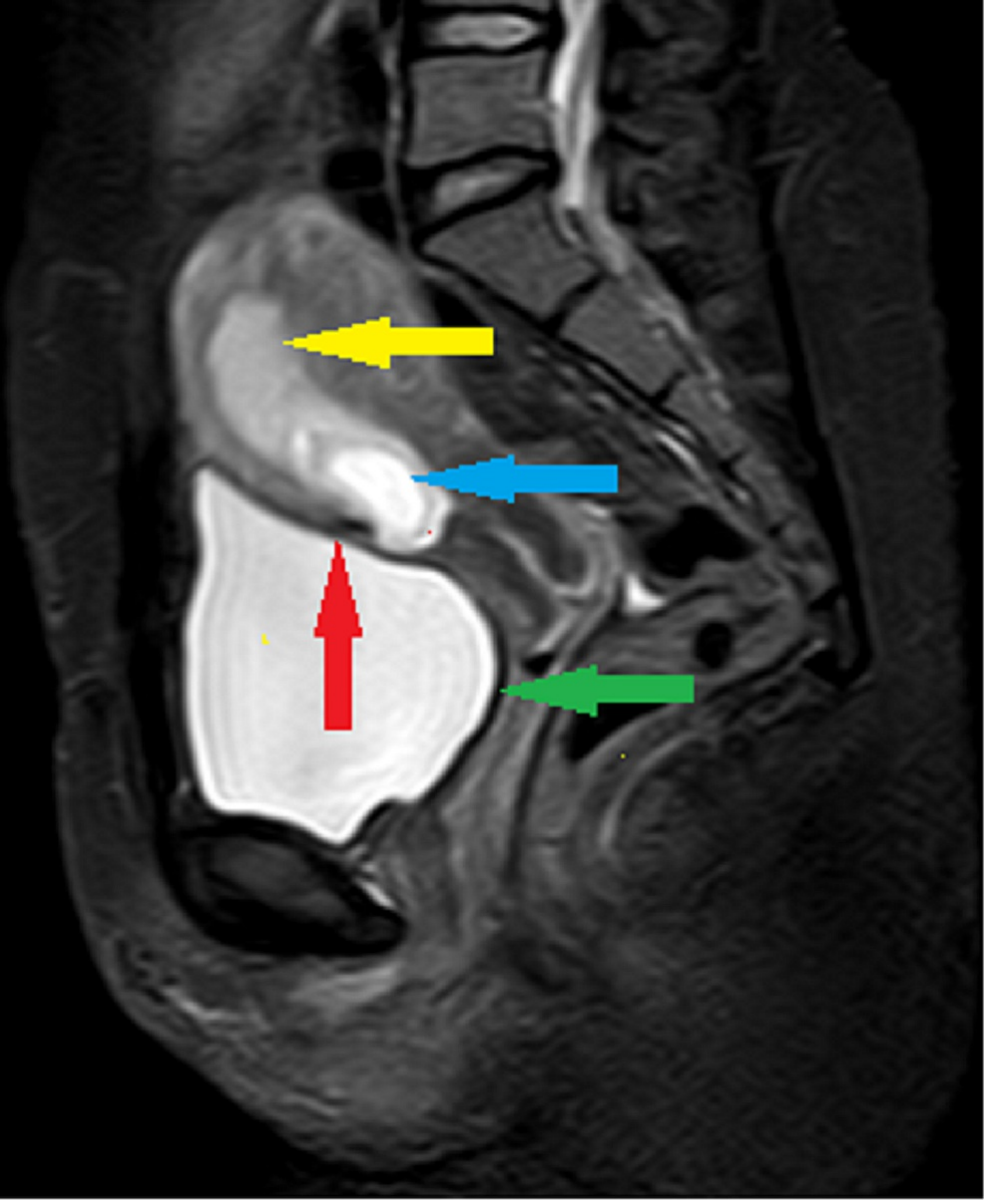
MRI pelvis T2W FAT-SAT sagittal image showing empty gestational sac located in lower uterine segment partially attached to the previous cesarean (blue arrow) within the endometrium (yellow arrow). The myometrium between the sac and bladder wall appears very thin (red arrow). Bladder wall appears intact and smooth (green arrow). T2W FAT-SAT, T2-weighted fat-saturation.

The patient got admitted to hospital for administration of injection methotrexate. Her β-hCG level on that day was 22,657 mIU/mL. Urgent MRI pelvis was done for confirmation of diagnosis and also to exclude viable pregnancy. MRI showed 26 mm oval gestational sac located in lower uterine segment partially attached to the scar of the previous cesarean section on the endometrial cavity side and extends toward the uterine cavity, the myometrium between the sac and bladder wall appears very thin and bladder wall was not disrupted, no clear yolk sac and embryo were seen, uterine cavity of upper segment was empty (Figure 2), features highly suggestive of cesarean scar ectopic pregnancy of endogenic type. So, the team proceeded with administration of injection methotrexate, to be given in four doses of 1 mg/kg body weight, on alternate days and alternating with injection folinic acid in doses of 0.1 mg/kg body weight. The patient agreed to take only two doses of methotrexate out of fear of its side effects. After receiving two doses on alternate days, she was discharged home for an elective surgical management. A week after, her serial β-hCG levels showed initial rise and then declined to 35,274 and 33,324 mIU/mL, respectively. She was admitted for surgical evacuation. She received vaginal prostin pessary and intrauterine Foley catheter for cervical ripening. She underwent uneventful suction evacuation of product of conception under ultrasound guidance, product removed with Suction Cannula 8 completely. After that, diagnostic hysteroscopy was done to check the integrity of the uterus, which showed cesarean scar intact with no active bleeding and no retained product in endometrial cavity. Post-procedure ultrasound also showed empty uterine cavity. The patient had uneventful post-procedure recovery and was discharged home on the same day.

Differential diagnosis

One of the crucial steps is to diagnose the cesarean ectopic pregnancy correctly. Its differential diagnosis includes a normally sited intrauterine pregnancy and a cervical pregnancy as the management of each type differs drastically. Transabdominal ultrasound in our patient fulfilled the following diagnostic criteria as described in the Green-top Guideline (GTG) of Royal College of Obstetrics and Gynecology: Diagnosis and Management of Ectopic Pregnancy GTG 21 [4].

Empty uterine cavity [5] with a gestational sac or solid mass of trophoblast located anteriorly at the level of internal is embedded at the site of previous lower segment cesarean section [6]. Thin or absent layer of myometrium between bladder and gestational sac is present [7]. There is sonographic evidence of prominent trophoblast/placental circulation on color Doppler imaging [8] and presence of empty endocervical canal [9].

An empty cervical canal and closed cervix in our patient excludes the cervical pregnancy, whereas empty uterine cavity particularly upper segment rules out intrauterine normally sited pregnancy.

Outcome and follow-up

The patient had an uneventful recovery. She was followed up after two weeks from surgery and was in good health. Histopathology report of the product of conception excluded any other pathology.

## Discussion

Ectopic pregnancy is the leading cause of morbidity and mortality in fertile women and is related to 4% of pregnancy-associated deaths [8]. One of the types of ectopic pregnancy is cesarean scar ectopic pregnancy in which the pregnancy implants onto the scar in uterus.

There are many theories that explain the pathogenesis of cesarean ectopic pregnancy. One theory is that blastocyst may implant in a microscopic dehiscent tract in a scar in the uterus. This scar may be of a cesarean section, any other uterine surgery like myomectomy [9], or even manual removal of placenta [10]. Another theory of intramural implantation is after in vitro fertilization and embryo transfer even in absence of uterine surgery history [11]. A uterine scar is deficient in decidua basalis or contains a faulty layer of fibrinoid degeneration. This ectopic pregnancy is not surrounded by decidualized endometrium; in fact, it implants into fibroid scar tissue and myometrium. This pregnancy is abnormal from the start and requires specialized management.

Most of the cesarean scar ectopic pregnancies are asymptomatic. Few can present with light vaginal bleeding or mild abdominal pain [12]. There are no pathognomonic signs or symptoms of cesarean scar ectopic pregnancy. Transvaginal ultrasound can be supplemented with transabdominal ultrasound as the main modality for diagnosis based on the criteria described in GTG 21 [4].

As it is a rare diagnosis, most of the evidence for management comes from case reports and small case series. Recent research supports any method that removes the pregnancy and scar to reduce morbidity and improve fertility [13]. Transabdominal and transvaginal approaches are employed; however, research is still required to identify the optimal approach. Key to optimal management is early termination of pregnancy with multidisciplinary approach.

There are two main modalities of management, medical and surgical. Medical management with injection methotrexate has been used effectively for cesarean scar pregnancies as in other types of ectopic pregnancies. Symptom-free, hemodynamically stable, unruptured pregnancy of gestational age < eight weeks are candidates for methotrexate [14]. It stops cells from dividing. It can be given as intrasac or local injection, systemic or intramuscular, or a combination of both. Administration of a single injection of methotrexate, potassium chloride (KCL), hyperosmolar glucose, or crystalline trichosanthin under ultrasound guidance has been used [6]. Reassuring results have been reported with systemic regimens, with and without intrasac medication injections, for cesarean scar pregnancy. Both single-dose and multidose protocols have been used. The standard single-dose regimen for methotrexate is 50 mg/m^2^, whereas the multidose protocol includes four doses of methotrexate 1 mg/kg given on Days 1, 3, 5, and 7 with alternating days of Folinic Acid 0.1 mg/kg [12]. Patients with ectopic pregnancies and hCG levels < 5,000 mIU/mL appear to respond best to systemic methotrexate. Close follow-up is required and may need to be combined with surgical approaches either electively or emergently if heavy bleeding starts.

Surgical management options include hysteroscopic suction evacuation and curettage, laparoscopic or open removal of scar along with pregnancy, and hemostatic measures including double balloon catheter for tamponade and uterine artery embolization. A number of authors advocate surgical approach even in absence of active bleeding [10]. It involves laparotomy with removal of scar and resuturing so as to reduce recurrence and limit follow-up period. Uterine curettage as first-line management is discouraged as it may ensue bleeding and uterine rupture or may fail to reach the product of conception [14]. However, it can be employed in combination with hysteroscopy under direct visualization and particularly after successful medical management. Transvaginal approach can be combined with laparoscopic approach. In a case series by Ash et al., hysteroscopy was used in conjunction with laparoscopy [15]. If anterior myometrial thickness was < 3 mm on ultrasound, laparoscopy was performed prior to hysteroscopy to dissect bladder peritoneum from the lower uterine segment to attempt to remove the bladder from the site of surgical management and decrease risk of injury. In this case series, 44 patients were successfully treated with removal of products of conception [16].

Other surgical options include uterine artery embolization, where embolus can be injected into uterine arteries prior to hysteroscopy to reduce the risk of bleeding. However, as this procedure may cause inadvertent ovarian embolization, patients’ plans for future fertility should be considered. Furthermore, double balloon catheter also can be used to tamponade the bleeding.

## Conclusions

Intramural pregnancy with implantation in a previous cesarean section scar is probably the rarest location for ectopic pregnancy. This type of pregnancy may become complicated with uterine rupture and life-threatening hemorrhage. Therefore, early diagnosis of cesarean scar ectopic gestation using sonography combined with Doppler flow imaging is of paramount importance, followed by confirmation of pelvic MRI if and when indicated. Although expectant management has been attempted in some cases, currently available data support termination of such a pregnancy once the correct diagnosis is made.
